# Once-Weekly Semaglutide Use in Patients with Type 2 Diabetes: Results from the SURE Spain Multicentre, Prospective, Observational Study

**DOI:** 10.3390/jcm11174938

**Published:** 2022-08-23

**Authors:** Virginia Bellido, Cristina Abreu Padín, Andrei-Mircea Catarig, Alice Clark, Sofía Barreto Pittol, Elias Delgado

**Affiliations:** 1 Unidad de Gestión Clínica de Endocrinología y Nutrición, Hospital Universitario Virgen del Rocío, 41013 Sevilla, Spain; 2 Instituto de Biomedicina de Sevilla (IBiS), Hospital Universitario Virgen del Rocío, CSIC, Universidad de Sevilla, 41013 Sevilla, Spain; 3 Hospital General de Segovia, 47002 Segovia, Spain; 4 Novo Nordisk A/S, DK-2760 Søborg, Denmark; 5 Novo Nordisk Pharma SA, 28033 Madrid, Spain; 6 Department of Endocrinology and Nutrition, Hospital Universitario Central de Asturias (HUCA), 33011 Oviedo, Spain; 7 Department of Medicine, University of Oviedo, 33006 Oviedo, Spain; 8 Health Research Institute of the Principality of Asturias (ISPA), 33011 Oviedo, Spain; 9 Spanish Biomedical Research Network in Rare Diseases (CIBERER), 28029 Madrid, Spain

**Keywords:** body weight, glucagon-like peptide-1 receptor agonist, HbA_1c_, real-world evidence, semaglutide, SURE study, type 2 diabetes

## Abstract

Type 2 diabetes (T2D) is a complex disease for which an individualised treatment approach is recommended. Once-weekly (OW) semaglutide is a glucagon-like peptide-1 receptor agonist approved for the treatment of insufficiently controlled T2D. The aim of this study was to investigate the use of OW semaglutide in adults with T2D in a real-world context. SURE Spain, from the 10-country SURE programme, was a prospective, multicentre, open-label, observational study, approximately 30 weeks in duration. Adults with T2D and ≥1 documented HbA_1c_ value ≤12 weeks before semaglutide initiation were enrolled. Change in HbA_1c_ from baseline to end of study (EOS) was the primary endpoint, with change in body weight (BW), waist circumference, and patient-reported outcomes as secondary endpoints. Of the 227 patients initiating semaglutide, 196 (86.3%) completed the study on-treatment with semaglutide. The estimated mean changes in HbA_1c_ and body weight between baseline and EOS were −1.3%-points (95% confidence interval (CI) −1.51;−1.18%-points) and −5.7 kg (95% CI −6.36;−4.98 kg). No new safety concerns were identified. Therefore, in routine clinical practice in Spain, OW semaglutide was shown to be associated with statistically significant and clinically relevant reductions in HbA_1c_ and BW in adults with T2D.

## 1. Introduction

Type 2 diabetes (T2D) places a heavy burden on individuals and healthcare systems across the world. In Spain, an estimated 13.8% of people have T2D, and this is expected to increase in the future [1,2].

The management of T2D is complex. The American Diabetes Association (ADA) Standards of Medical Care in Diabetes 2022 [3] and the 2020 joint consensus statement of the ADA and the European Association for the Study of Diabetes (EASD) [4] recommend that physicians should take an individualised treatment approach when prescribing medications for T2D, and that they consider drug efficacy, risk of hypoglycaemia, cardiorenal benefits, effect on body weight (BW), adverse effects, pricing, and convenience for the patient [4].

Glucagon-like peptide-1 receptor agonists (GLP-1RAs) are an established class of antihyperglycaemic drugs used for the treatment of T2D, which have demonstrated improvements in glycaemic control and reductions in BW in patients with T2D [5,6]. In addition to their glucose-dependent function resulting in a low risk for hypoglycaemia [7], some GLP-1RAs (dulaglutide, liraglutide, and semaglutide) have demonstrated cardiovascular (CV) benefits in patients with T2D at high risk of CV disease [8,9,10]. Despite these benefits, access to GLP-1RAs is limited in Spain, and GLP-1RAs are only reimbursed for patients with obesity (body mass index [BMI] ≥ 30 kg/m^2^) and insufficient glycaemic control as a second-line therapy after metformin [11].

Semaglutide is a human GLP-1 analogue, approved as an add-on to diet and exercise for the treatment of adults with insufficiently controlled T2D, by the European Medicines Agency in February 2018 [12]. It has a long half-life, which makes it suitable for once-weekly (OW) dosing, [13] and is the only GLP-1RA that is available both in a OW subcutaneous (s.c.) injectable formulation and as an oral formulation administered once-daily [14].

The extensive SUSTAIN randomised clinical trial (RCT) programme, which investigated the efficacy and safety of OW s.c. semaglutide, demonstrated that 0.5 mg and 1.0 mg doses were associated with superior, clinically relevant improvements in glycaemic control and weight loss, compared with placebo or active comparators [8,15,16,17,18,19,20,21,22,23]. A safety profile similar to other GLP-1RAs was also observed.

SURE Spain is part of the SURE real-world study programme, which aimed to explore the use of OW semaglutide in a diverse population of adults with T2D in routine, real-world clinical practice across 10 countries (Canada, Denmark/Sweden, France, Germany, Italy, the Netherlands, Spain, Switzerland, and the United Kingdom) and to complement the results of the SUSTAIN RCTs. Unlike RCTs, the SURE studies are non-interventional and observational, allowing the assessment of patient outcomes, as well as product use and performance, in diverse patient populations in routine clinical practice [24].

The aim of this study was to evaluate the real-world use of OW semaglutide in a diverse T2D patient population in Spain.

## 2. Materials and Methods

### 2.1. Study Design

SURE Spain was a multicentre, prospective open-label, single-arm, non-interventional study assessing the use of OW semaglutide in adult patients with T2D in routine clinical practice in Spain. Informed consent and treatment initiation took place on the first visit (week 0), followed by an anticipated exposure period of ~30 weeks (range: 28–38 weeks). Intermediate visits scheduled according to local practice and data collection were performed throughout the entire study.

The decision to initiate semaglutide treatment was at the discretion of the treating physician, following requirements stated in the Summary of Product Characteristics (SmPC), therapeutic positioning report and local/regional guidelines, and clearly separated from the decision to include the patient in the SURE Spain study. All parameters collected in the study (except the patient-reported outcomes) were part of routine clinical practice. Patients were treated OW with commercially available s.c. semaglutide (Ozempic^®^; Novo Nordisk A/S, Bagsværd, Denmark), available in a pre-filled, multidose, pen injector. The treating physician determined the maintenance dose and any subsequent changes to it. Diet and physical activity counselling could be offered in line with routine clinical practice, with modifications to prescribed antihyperglycaemic treatment at the physician’s discretion.

This study was conducted in accordance with the Declaration of Helsinki [25], the Guidelines for Pharmacovigilance Practices Module VI [26], and Good Pharmacoepidemiology Practices [27]. Prior to study initiation, the protocol, protocol amendment, patient information/informed consent form, together with any other written information to be provided to the patient and patient enrolment procedures, were reviewed and approved by the independent ethics committee/institutional review board at each study site (first approved in 2019 by the Ethics Committee of CEIm de EUSkadi, project identifier: NN9535-4368). Written informed consent was obtained from all patients prior to any study-related activities. This study is registered on ClinicalTrials.gov (NCT04067999).

### 2.2. Study Population

Adult patients (age ≥ 18 years) diagnosed with T2D were included from 34 sites in Spain, with the first participant’s first visit on 5 August 2019, and the last participant’s last visit on 19 July 2021. Inclusion criteria included diagnosis of T2D and availability of one or more documented values of HbA_1c_ within 12 weeks prior to semaglutide treatment initiation. Exclusion criteria included previous participation in a SURE study, mental incapacity, unwillingness, or language barriers precluding adequate understanding or cooperation, prior treatment with any investigational drug (90 days before enrolment), and hypersensitivity to semaglutide or any of the excipients. The study duration of 30 weeks was considered sufficient to initiate and optimise the study treatment regimen and to obtain real-world data for the evaluation of the primary endpoint.

### 2.3. Endpoints

The primary endpoint was a change from baseline to end of study (EOS) in HbA_1c_ (%-point and mmol/mol). Supportive secondary endpoints included: change from baseline to EOS in BW (kg and %) and waist circumference (cm); proportion of patients achieving HbA_1c_ < 8.0% (64 mmol/mol), <7.5% (59 mmol/mol) and <7.0% (53 mmol/mol) [28]; reduction in HbA_1c_ from baseline to EOS of ≥1.0%-point; weight reduction from baseline to EOS of ≥3.0% [29] and ≥5.0%; HbA_1c_ reduction from baseline to EOS of ≥1.0% and weight reduction from baseline to EOS of ≥3.0% [29]; patient-reported severe or documented hypoglycaemia between baseline and EOS; and change from baseline to EOS in scores for patient-reported outcomes of: the Diabetes Treatment Satisfaction Questionnaire–status (DTSQs; absolute treatment satisfaction) comprising eight questions, of which six questions are combined into a total Treatment Satisfaction score (scale: 0 to 36); the Diabetes Treatment Satisfaction Questionnaire–change (DTSQc; relative treatment satisfaction), total treatment satisfaction (scale: −18.0 to 18.0); and the 36-item Short-Form Health Survey version 2 (SF-36^®^v2), physical and mental summary component. The proportion of patients who completed the study under treatment with semaglutide was also investigated.

Exploratory assessments included: weekly dose of semaglutide at EOS; proportion of patients who had not added new antihyperglycaemic drug(s) to semaglutide treatment at any time during the study, evaluated at EOS; proportion of patients who had achieved clinical success, in relation to the reason to initiate semaglutide treatment, as assessed by the physician at EOS; patient-reported 8-Item Morisky Medication Adherence Scale (MMAS-8) score at EOS (low, medium, high) [30,31,32]; and the number of severe or documented hypoglycaemic episodes. Post hoc assessments included change from baseline to EOS in BMI (kg/m^2^). Permission for use of the MMAS-8 was granted prior to the study.

### 2.4. Safety

Only information on serious adverse drug reactions (SADRs), fatal events, pregnancies in female patients, and adverse events (AEs) in foetuses or newborns were systematically collected during the study. Voluntary reporting of other safety information by the physician followed the same process as for the systematic safety reporting. All episodes of patient-reported documented and/or severe hypoglycaemia were to be recorded.

### 2.5. Statistical Analyses

Power calculations showed that a sample size of 130 patients was required, based on the criterion of 90% probability of obtaining a 95% confidence interval (CI) for mean change from baseline in HbA_1c_ whose half-width was at most 0.30. The half-width of 0.30 was chosen as a reasonable uncertainty allowing for a robust evaluation of glycaemic efficacy, in line with diabetes guidelines [33]. To ensure sufficient statistical power to evaluate the efficacy of semaglutide on glycaemic control (on the basis of evidence from previous observational studies with GLP-1RA treatment), it was necessary to include at least 217 enrolled patients initiating semaglutide, to ensure that 130 patients completed the study on-treatment [34,35].

The Full Analysis Set (FAS), which included all patients in the study who initiated semaglutide treatment, was used for characterising baseline demographics, analysis of the secondary endpoint related to study completion on-treatment, the selected exploratory assessments, description of AEs, and the sensitivity analyses of the primary and secondary endpoints.

The Effectiveness Analysis Set (EAS) included all patients in the FAS who completed the study and were receiving semaglutide treatment at EOS. The EAS was used for characterising baseline demographics at EOS, the description of antihyperglycaemic medications at baseline and EOS, and the primary, secondary and exploratory endpoint analyses.

Baseline demographic data are summarised using descriptive statistics (mean ± standard deviation [SD] or median and interquartile range for continuous variables and number and proportion for categorical variables). Change in the continuous variables of the primary and secondary endpoints from baseline to EOS were analysed using the Analysis of Covariance (ANCOVA) model. Categorical endpoints were analysed using descriptive statistics.

Sensitivity analyses investigated the robustness of the conclusions from the main analyses and explored the impact of missing data in the primary analysis, for which patients were excluded if they did not complete the study or discontinued treatment, or if HbA_1c_ data were missing at EOS. The prespecified in-study sensitivity analysis of the primary endpoint included all patients in the FAS with at least one post-baseline HbA_1c_ measurement in the in-study period. For this analysis, the primary endpoint was analysed using a Mixed Model for Repeated Measures (MMRM) including all HbA_1c_ assessments in the in-study period. The on-treatment sensitivity analysis included patients in the FAS with at least one post-baseline HbA_1c_ assessment, but it only included HbA_1c_ assessments in the on-treatment period and used the same statistical approach as the in-study sensitivity analysis.

Because of the COVID-19 pandemic, the EOS visit (V6) window was extended beyond 38 weeks to allow participants to complete their EOS assessments. Consequently, an additional post hoc sensitivity analysis was performed to explore the impact of extending the EOS visit (V6) window on the primary endpoint. The sensitivity analysis of the primary endpoint was the same as the primary analysis of the primary endpoint but included only those patients who had an EOS visit between weeks 28 and 38 (the original visit window). An additional post hoc sensitivity analysis was performed to explore the impact of extending the EOS visit on the secondary endpoint of change from baseline to EOS in BW. This sensitivity analysis was the same as the main analysis of this endpoint but included only those patients who had an EOS visit between weeks 28 and 38.

## 3. Results

### 3.1. Patient Population and Baseline Characteristics

**Figure 1:**
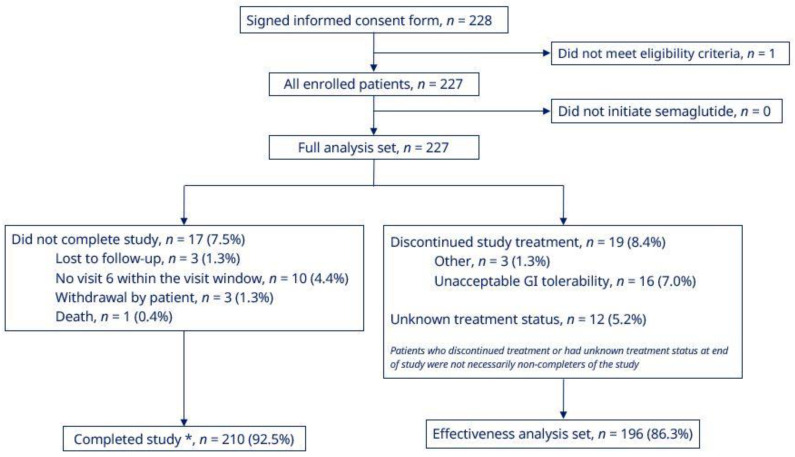
Patient disposition. * Patients who initiated semaglutide treatment and attended the end of study visit. GI, gastrointestinal.

Of the 228 patients who signed the consent form, one did not meet the eligibility criteria. Therefore, the FAS comprised the 227 patients who were enrolled in the study and who had initiated semaglutide treatment (Figure 1). A total of 210 patients (92.5%) completed the study, and the mean treatment duration was 33.7 weeks. The reasons for non-completion were: death (*n* = 1; 0.4%), lost to follow-up (*n* = 3; 1.3%), withdrawal by patient (*n* = 3; 1.3%), and missed EOS visit within the visit window (*n* = 10; 4.4%) (Figure 1). The EAS comprised 196 patients (86.3%) who had completed the study on semaglutide treatment (Figure 1). Twelve patients (5.2% of the FAS) had an unknown treatment status at EOS. With regard to discontinuations, 16 patients (7.0%) discontinued treatment due to unacceptable gastrointestinal (GI) intolerability, and a further three patients (1.3%) had ‘other’ recorded as the reason (Figure 1).

Baseline characteristics of patients are summarised in Table 1. Hypertension and dyslipidaemia were the most frequent CV comorbidities at baseline, affecting 75.8% and 76.2% of patients, respectively.

Most patients initiated semaglutide at a dose of 0.25 mg (83.3%); 13.7% initiated at 0.5 mg and 3.1% at 1.0 mg. The most common reasons for initiating semaglutide as part of T2D treatment were weight reduction (94.3%) and to improve glycaemic control (88.5%).

The most frequent antihyperglycaemic drugs used by patients in the EAS at baseline were metformin (75.5% of patients), sodium–glucose cotransporter-2 inhibitors (SGLT-2is) (42.3%), basal insulin (32.7%), and dipeptidyl peptidase-4 inhibitors (DPP-4is) (20.4%) (Supplementary Table S1).

### 3.2. HbA_1c_, BW, BMI, and Waist Circumference Outcomes

For patients in the EAS receiving semaglutide, statistically significant reductions were observed at EOS for mean HbA_1c_, BW, waist circumference and BMI (Table 2). The mean HbA_1c_ at EOS was 7.1%, and the estimated mean change from baseline was −1.3%-points [95% CI −1.51;−1.18%-points; *p* < 0.0001] (Table 2, Supplementary Figure S1); mean BW at EOS was 93.2 kg, and the estimated mean change from baseline was −5.7 kg [95% CI −6.36; −4.98 kg; *p* < 0.0001] (Table 2, Supplementary Figure S1); mean BMI at EOS was 34.4 kg/m^2^_,_ and the estimated mean change from baseline was −2.1 kg/m^2^ [95% CI −2.37; −1.86 kg/m^2^; *p* < 0.0001]; and mean waist circumference at EOS was 113.4 cm, and the estimated mean change from baseline to EOS was −5.3 cm [95% CI −6.29; −4.41 cm; *p* < 0.0001] (Table 2, Supplementary Figure S1).

**Figure 2:**
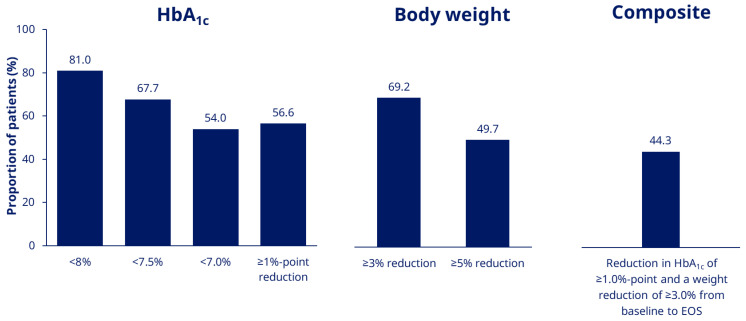
Proportion of patients achieving HbA_1c_ targets and weight-loss goals (EAS). EAS, Effectiveness Analysis Set; EOS, end of study.

At EOS, 81.0%, 67.7% and 54.0% of patients in the EAS had an HbA_1c_ of < 8.0%, <7.5% and <7.0%, respectively (Figure 2). The proportion of patients achieving an HbA_1c_ reduction ≥1%-point was 56.6% and the proportions achieving weight reduction of ≥ 3.0% and ≥5.0% were, respectively, 69.2% and 49.7% (Figure 2). The proportion of patients in the EAS achieving the composite endpoint of an HbA_1c_ reduction of ≥ 1.0% and weight reduction ≥3.0% at EOS was 44.3% (Figure 2). In the FAS, 86.3% of patients completed the study on-treatment with semaglutide (Figure 1).

### 3.3. Sensitivity Analyses

Prespecified sensitivity analyses were used to explore the impact of missing data in the main analysis. The on-treatment sensitivity analysis of the FAS showed that the mean HbA_1c_ decreased over time from initiation of semaglutide to week 30, with an estimated change of −1.4%-points [95% CI −1.59; −1.27%-points] (Supplementary Figure S2). The estimated mean changes from baseline to EOS and associated 95% CIs were similar across sensitivity analyses and showed that the mean changes in HbA_1c_ were statistically significantly different from having no mean change in HbA_1c_ (Supplementary Table S2, Supplementary Figure S2). Moreover, the estimated mean HbA_1c_ and estimated change in HbA_1c_ were similar over the course of the study for both the in-study and on-treatment period.

Additional post hoc sensitivity analyses were performed in patients who had their EOS visit within the original visit window (week 28–38). The post hoc analyses of the mean changes from baseline to EOS for HbA_1c_ and for BW showed similar results to those seen in the primary analysis (Supplementary Table S3).

Collectively, the sensitivity analyses supported the conclusions from the primary analysis, which included assessments for patients who were on-treatment at the EOS visit (V6), also including those completing the study after week 38.

### 3.4. Semaglutide Dose

The mean ± SD weekly dose of semaglutide at EOS was 0.85 ± 0.24 mg. At EOS, five (2.6%) patients were receiving 0.25 mg OW semaglutide, 50 (25.5%) were receiving 0.5 mg, two (1.0%) were receiving between >0.5 mg and <1.0 mg, and 139 (70.9%) were receiving 1.0 mg.

### 3.5. Patient-Reported Outcomes

**Figure 3:**
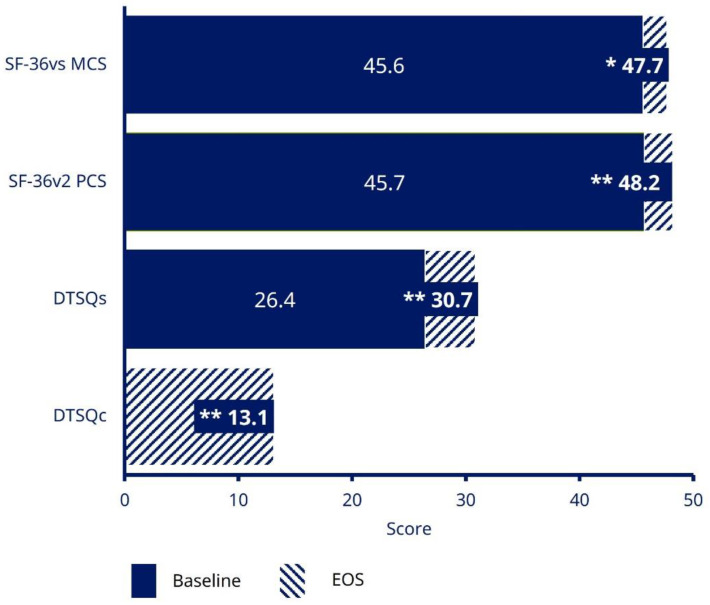
Treatment satisfaction and HRQoL (EAS). * *p* = 0.0013; ** *p* < 0.0001. Data are based on EAS. DTSQ status version (DTSQs) was measured at the informed consent and initiation visit, and the EOS visit; with responses ranging from 0 (very dissatisfied) to 6 (very satisfied) for each item of the questionnaire. The maximum total score is 36. The SF-36^®^v2 questionnaire has 36 questions grouped into eight domains, which can be combined into two summary component scores (overall mental and physical health); a higher SF-36^®^v2 score indicates lower disability. DTSQ, Diabetes Treatment Satisfaction Questionnaire; DTSQc, DTSQ change version; DTSQs, DTSQ status version; EAS, Effectiveness Analysis Set; EOS, end of study; HRQoL, health-related quality of life; MCS, mental component summary; PCS, physical component summary; SF-36^®^v2, 36-Item Short-Form Health Survey version 2.

In patients receiving semaglutide at EOS, DTSQs score increased by 4.4 [95% CI 3.66; 5.07; *p* < 0.0001] from baseline to EOS, representing a significant increase in absolute treatment satisfaction (Figure 3). Patients receiving semaglutide also reported a DTSQc score at EOS of 13.1 (95% CI 12.36; 13.85) out of a maximum score of 18, indicating a significant relative improvement in treatment satisfaction (*p* < 0.0001) (Figure 3).

At EOS, significant increases were observed in both the SF-36^®^v2 health-related quality-of life (HRQoL) questionnaire physical component score (*p* < 0.0001) and the mental component score (*p* = 0.0013), indicating an improvement in quality of life from baseline to EOS (Figure 3).

Mean MMAS-8 score was 7.0 at baseline and 7.4 at EOS, indicating a medium level of treatment adherence. The proportion of participants with medium and high adherence was, respectively, 49.5% and 36.6% at baseline and 34.1% and 57.3% at EOS.

### 3.6. Adverse Events and Hypoglycaemia

AEs and severe or documented hypoglycaemic episodes in patients receiving semaglutide are summarised in Table 3. In the FAS, 15 patients (6.6%) reported 26 treatment-emergent AEs: 88.5% of AEs were non-serious (13 [5.7%] patients; 23 events) and 46.2% were moderate in intensity (7 [3.1%] patients; 12 events). A total of 13 patients reported 22 GI AEs, which accounted for the highest number of AEs by system organ class. Three serious AEs (SAEs) were reported (Medical Dictionary for Regulatory Activities preferred terms: atrial fibrillation, left ventricular failure, and myocardial infarction) by two patients (0.9%), which were all judged as unlikely to be related to semaglutide treatment by the investigators. One severe SAE (preferred term: myocardial infarction) was reported, which had a fatal outcome.

Eight patients (4.1%) in the EAS reported severe or documented hypoglycaemia episodes between baseline and EOS, with similar results in the FAS (12 patients; 5.3%). At EOS, 20 severe or documented hypoglycaemic episodes were reported in the EAS, and 26 were reported in the FAS. Of these 26 events, 22 were reported by patients while using insulin and 3 occurred while using sulphonylureas. The date of one hypoglycaemic episode was unrecorded, which prevented an assessment of concurrent medication use. No severe hypoglycaemic episodes were reported during the study.

## 4. Discussion

The SURE Spain study is part of the SURE study programme, which consists of nine observational studies in ten countries and was conducted to assess the real-world use of OW semaglutide.

The data reported indicate that when OW s.c. semaglutide was taken according to local clinical practice by adult patients with T2D in Spain, a clinically relevant and statistically significant reduction, compared with baseline, was observed for HbA_1c_ at EOS (*p* < 0.0001) [36]. This was observed despite 14.5% of the study population switching from another GLP-1RA to semaglutide at baseline. While previous treatment may be expected to influence outcomes, improvements have been reported in patients treated with semaglutide who were not naïve to GLP-1RAs [36].

At EOS, patients also experienced statistically significant decreases from baseline in BW and waist circumference. A total of 97 (49.7%) patients achieved a weight reduction from baseline of ≥5%. This weight reduction is a key consideration in terms of reducing CV risk, in view of the beneficial reductions in triglycerides, total cholesterol, and low-density lipoprotein cholesterol that are associated with a weight loss of 5–10% [37].

Furthermore, patients reported substantial improvements in treatment satisfaction and HRQoL, as measured by the DTSQ and the SF-36^®^v2, respectively. In addition, patients’ adherence to OW semaglutide treatment was good, with 91.4% of patients reporting either high (57.3%) or medium (34.1%) adherence at EOS. Adherence to OW semaglutide in the SURE Spain study compares favourably to the adherence rates of 39.1–64.5% at 1 year reported for GLP-1RAs (including semaglutide) in retrospective, real-world cohort studies [37,38].

Additionally, the patient population in this study had advanced T2D, as indicated by the mean disease duration of 11.8 years from diagnosis and the complex pharmacological treatment at baseline, with 41.9% of patients taking more than two antihyperglycaemic medications and 47.5% taking insulin. These factors are associated with poorer treatment outcomes and make it more difficult to achieve treatment goals.

Drawing comparisons between GLP-1RA RCTs and real-world evidence studies from different countries can be challenging. Local T2D clinical guidelines vary and can restrict clinical access to GLP-1RAs, while local reimbursement policies may impose further barriers to patient access. In Spain, outside of private practice, GLP-1RAs are only reimbursed for patients with a BMI ≥ 30 kg/m^2^. This is in contrast with Denmark, where the recommendation is independent of BMI, and the UK, where use is recommended in those with a BMI ≥ 35 kg/m^2^ who show an adequate metabolic response.

Overall, the results of this study support previously reported data on the real-world use of OW semaglutide in Spain [39,40,41]. The reduction in HbA_1c_ and BW observed in Spain align with those observed in the countries that have published results from the SURE programme to date—Canada, Denmark/Sweden, Switzerland, and the UK—for which the mean change in HbA_1c_ from baseline to EOS was between −0.8 and −1.5%-points and the mean change in BW from baseline to EOS was between −4.3 and −5.8 kg [42,43,44,45]. The results are also aligned with real-world evidence from other countries, for example, a study by Marzullo et al., that showed reductions in HbA1c and body weight after 6 and 12 months of OW semaglutide treatment in people with T2D in Italy [46].

Metabolic control in patients with T2D is assessed using multiple factors (e.g., BW, waist circumference), and not only HbA_1c_. In SURE Spain, the majority (70.9%) of patients were receiving the recommended dose of 1.0 mg OW of semaglutide by EOS, and the significant improvements in primary and secondary endpoints in the study may indicate that this dose is appropriate for the goal of achieving global metabolic control. The safety findings of the real-world T2D population in Spain were also consistent with the safety profile of semaglutide established in the SUSTAIN programme and with that of the GLP-1RA class, with no unexpected safety issues reported.

### Study Limitations

The SURE Spain study was non-interventional and single-armed, so the potential impact of other predictive factors cannot be excluded. The fundamental limitation of such a study design is the absence of a randomised comparator, which would otherwise have enabled differentiation of the changes caused by treatment, and the impact of other factors. Data in this study were collected during routine clinical practice, rather than through mandated examinations at predetermined time points, which may have impacted the robustness and completeness of the dataset.

The primary analysis was based on data from patients who completed the study on semaglutide treatment and with their HbA_1c_ levels recorded at EOS. This could have resulted in selection bias, because patients who benefit from the study treatment are more likely to continue than those who do not. To account for this, sensitivity analyses of the primary endpoint included all post-baseline HbA_1c_ assessments as well as evaluations from intermediate visits also including patients who did not complete the study or discontinued semaglutide during the study. In addition, secondary supportive analyses assessed the percentage of patients who had started semaglutide treatment and were receiving it at the EOS.

The inclusion criteria were purposely designed to be broad and reflect a real-world T2D population, which is rarely the case in a standard RCT. However, it is likely that physicians who were highly motivated would have been overrepresented among the participating centres, and that the centres included either highly motivated patients or patients who were difficult to treat with the other therapies available. As a result, the enrolled group may only represent subsets of individuals who are eligible for semaglutide therapy. Nevertheless, study participants were profiled in terms of demographics and clinical data, which allowed for the assessment of the representativeness of the recruited population. Details of medical history (including T2D diagnosis) and concurrent diseases were obtained without further confirmation as provided by the investigators.

A potential limitation of SURE Spain is the study’s geographical location and time of initiation. The study was conducted soon after the launch of OW semaglutide, in a real-world setting, in a diverse T2D population recruited by investigators at 34 sites in Spain. However, the 34 sites that enrolled patients account for approximately half of the communities/regions within Spain, so may not be representative of the entire population. Furthermore, in Spain, GLP-1RAs are only reimbursed for patients who have a BMI ≥ 30 kg/m^2^, and only approximately 8% of Spanish patients with T2D are prescribed GLP-1RAs [11,47]. Therefore, none of the patients enrolled in the study had a ‘normal’ BMI (≥18.5–<25 kg/m^2^). These country-specific factors may have influenced the study results; in the future, however, semaglutide will likely be prescribed to a broader range of patients with T2D, including those with less severe disease progression.

The COVID-19 pandemic impacted intermediate and EOS visits in SURE Spain. Because of accessibility issues, several of these visits were instead conducted by telephone, rather than in-person. To further mitigate the challenges raised by the pandemic, changes were made to the study design that allowed patients to postpone their last visit (after the 38-week timepoint). An additional post hoc sensitivity analysis was performed to assess how the primary and secondary endpoints were affected by extending the EOS visit window. Extending the EOS visit window had no impact on the study outcomes.

Evidence has been reported that patients with T2D in Spain may have gained weight during the COVID-19 lockdown, due to their substantial lifestyle changes [48]. Sánchez et al. noted that if another lockdown were to be imposed, there should be greater emphasis on avoiding weight gain, in which case GLP-1RAs might be an effective therapy for these patients. Despite the influence of COVID-19, the data from this study are regarded as robust, and are suitable for further interpretation.

## 5. Conclusions

In SURE Spain, patients treated with OW semaglutide experienced statistically significant and clinically relevant reductions from baseline to EOS in HbA_1c_, BW, and waist circumference, and improvements in other clinical parameters such as treatment satisfaction and HRQoL in a real-world setting. These findings were significant, despite the nature of the population (advanced T2D) included in the SURE Spain study and the local limitations on prescribing GLP-1RAs. The reported AEs were consistent with the known safety profile of semaglutide, with no new safety concerns reported. These results support the use of OW semaglutide in routine clinical practice in adults with T2D in Spain.

## Figures and Tables

**Table 1:** Baseline characteristics of patients (FAS).

**N**	227
Age, years	59.1 (9.94)
Female, *n* (%)	111 (48.9)
Race, *n* (%)	
White	221 (97.4)
American Indian or Alaska Native	2 (0.9)
Other	4 (1.8)
Body weight, kg	98.3 (17.89)
Waist circumference, cm	118.8 (12.50)
BMI, kg/m^2^	36.4 (5.28)
BMI categories, *n* (%)	
Normal (18.5−<25 kg/m^2^)	0
Overweight (25−<30 kg/m^2^)	12 (5.3)
Obese class I (30−<35 kg/m^2^)	89 (39.6)
Obese class II & III (≥35 kg/m^2^)	124 (55.1)
Diabetes duration, years	11.8 (8.10)
Baseline HbA_1c_, %	8.5 (1.58)
HbA_1c_ level, *n* (%)	
<8.0%	93 (41.0)
<7.5%	62 (27.3)
<7.0%	34 (15.0)
Baseline HbA_1c_, mmol/L	69.1 (17.3)
FPG, mmol/L	9.9 (3.46)
eGFR, mL/min/1.73 m^2^	82.4 (22.58)
Lipid composition, mg/dL	
HDL cholesterol	44.8 (13.31)
LDL cholesterol	92.5 (30.76)
Total cholesterol	175.7 (45.41)
Triglycerides	243.9 (298.8)
Lipid composition, mmol/L	
HDL cholesterol	1.2 (0.34)
LDL cholesterol	2.4 (0.80)
Total cholesterol	4.6 (1.18)
Triglycerides	2.8 (3.37)
Comorbid conditions at baseline, *n* (%)	
Diabetic retinopathy	29 (12.9)
Diabetic neuropathy	18 (7.9)
Diabetic nephropathy	38 (16.7)
Dyslipidaemia	173 (76.2)
Hypertension	172 (75.8)

Values based on FAS (*n* = 227). Data for continuous variables are mean (SD) unless otherwise specified. BMI, body mass index; eGFR, estimated glomerular filtration rate; FAS, Full Analysis Set; FPG, fasting plasma glucose; HDL, high-density lipoprotein; LDL, low-density lipoprotein; SD, standard deviation.

**Table 2:** Change from baseline to EOS in HbA_1c_, body weight, waist circumference, and BMI (EAS).

	N	*n*	Estimate	95% CI	*p*-Value
**HbA_1c_, %**	**196**	**187**	**-**	**-**	**-**
Observed mean at baseline	-	-	8.4	-	-
Estimated mean at EOS	-	-	7.1	-	-
Change from baseline to EOS	-	-	−1.3	[−1.51; −1.18]	<0.0001
**HbA_1c_, mmol/mol**	**196**	**187**	**-**	**-**	**-**
Observed mean at baseline	-	-	68.5	-	-
Estimated mean at EOS	-	-	53.8	-	-
Change from baseline to EOS	-	-	−14.7	[−16.48; −12.86]	<0.0001
**Body weight, kg**	**196**	**194**	**-**	**-**	**-**
Observed mean at baseline	-	-	98.9	-	-
Estimated mean at EOS	-	-	93.2	-	-
Change from baseline to EOS	-	-	−5.7	[−6.36; −4.98]	<0.0001
Percent change from baseline to EOS	-	-	−5.7	[−6.41; −5.03]	<0.0001
**Waist circumference, cm**	**196**	**165**	**-**	**-**	**-**
Observed mean at baseline	-	-	118.8	-	-
Estimated mean at EOS	-	-	113.4	-	-
Change from baseline to EOS	-	-	−5.3	[−6.29; −4.41]	<0.0001
**BMI, kg/m^2^**	**196**	**194**	**-**	**-**	**-**
Observed mean at baseline	-	-	36.5	-	-
Estimated mean at EOS	-	-	34.4	-	-
Change from baseline to EOS	-	-	−2.1	[−2.37; −1.86]	<0.0001

Data are based on the EAS, which included patients who attended the EOS visit and were still receiving semaglutide. Change in response from baseline to EOS is analysed using baseline, T2D duration, age, BMI, pre-initiation use of GLP-1RA, pre-initiation use of DPP-4i, pre-initiation use of insulin, number of OADs used pre-initiation (0–1/2+) and sex as covariates. *p*-value is reported for no average change in response from baseline to EOS. The assessment of BMI was performed as a post hoc analysis. BMI, body mass index; CI, confidence interval; DPP-4i, dipeptidyl peptidase-4 inhibitor; EAS, Effectiveness Analysis Set; EOS, end of study; GLP-1RA, glucagon-like peptide-1 receptor agonist; N, total number of patients in EAS; *n*, total number of patients included in analyses; OAD, oral antihyperglycaemic drug; T2D, type 2 diabetes.

**Table 3:** AEs and severe or documented hypoglycaemic episodes in patients receiving semaglutide (FAS).

	Serious			Non-Serious			Total		
	N	(%)	E	N	(%)	E	N	(%)	E
AE	2	0.9	3	13	5.7	23	15	6.6	26
Severity									
Mild	0	0	0	6	2.6	12	6	2.6	12
Moderate	1	0.4	2	6	2.6	10	7	3.1	12
Severe	1	0.4	1	1	0.4	1	2	0.9	2
GI disorders	0	0	0	13	5.7	22	13	5.7	22
Nausea	0	0	0	7	3.1	7	7	3.1	7
Vomiting	0	0	0	5	2.2	5	5	2.2	5
Diarrhoea	0	0	0	3	1.3	3	3	1.3	3
AEs leading to treatment discontinuation	0	0	0	5	2.2	6	5	2.2	6
SADRs	0	0	0	0	0	0	0	0	0
							**N**	(**%**)	
**Patients with severe or documented hypoglycaemic episodes**							**8**	**4.1**	

All other events were reported on a voluntary basis (FAS). AE, adverse event; E, event; FAS, Full Analysis Set; GI, gastrointestinal; N, total number of patients in FAS; SADR, serious adverse drug reaction.

## Data Availability

The datasets analysed during the current study are available from the corresponding author on reasonable request.

## Supplementary Materials

The following supporting information can be downloaded at: https://www.mdpi.com/article/10.3390/jcm11174938/s1.
