# Supplementary material for: Once-Weekly Semaglutide Use in Patients with Type 2 Diabetes: Results from the SURE Spain Multicentre, Prospective, Observational Study
Source: J Clin Med. 2022 Aug 23;11(17):4938. doi: 10.3390/jcm11174938 (PMC9456474; doi:10.3390/jcm11174938)
Supplement: Supplementary file 1 [file jcm-11-04938-s001.zip › jcm-1805710-supplementary.pdf]

Supplementary material

Supplementary Table S1: Antihyperglycaemic medications at baseline and EOS (EAS)

**Medication type, n (%)**	**Baseline (N=196)**	**EOS (N=196)**
Metformin	148 (75.5)	149 (76.0)
SUs	23 (11.7)	12 (6.1)
Combinations of oral blood glucose lowering drugs	8 (4.1)	7 (3.6)
TZDs	1 (0.5)	2 (1.0)
DPP-4is	40 (20.4)	7 (3.6)
SGLT-2is	83 (42.3)	87 (44.4)
Other GLP-1RAs	32 (16.3)	-
Basal insulin	64 (32.7)	71 (36.2)
Premixed insulin	9 (4.6)	8 (4.1)
Fast-acting insulin	20 (10.2)	18 (9.2)
Total receiving insulin	93 (47.5)	97 (49.5)
Other blood glucose lowering drugs, excluding insulins	13 (6.6)	10 (5.1)
No medication	1 (0.5)	9 (4.6)
Number of antihyperglycaemic medications		
0	1 (0.5)	0
1	50 (25.5)	9 (4.6)
2	63 (32.1)	60 (30.6)
>2	82 (41.9)	127 (64.8)

DPP-4i, dipeptidyl peptidase 4 inhibitor; EAS, Effectiveness Analysis Set; EOS, end of study; GLP-1RA, glucagon-like peptide-1 receptor agonist; SGLT-2i; sodium–glucose co-transporter-2 inhibitor; SU, sulphonylurea; TZD, thiazolidinedione.

Supplementary Table S2: Prespecified sensitivity analyses of the change from baseline to EOS in HbA_{1c} (FAS)

	N	n	Estimate	95% CI	P-value
**In-study sensitivity analysis**					
**HbA_{1c}, %**	**227**	**218**	**-**	**-**	**-**
Observed mean at baseline	-	-	8.4	-	-
Estimated mean at week 30	-	-	7.1	-	-
Change from baseline to week 30, %-point	-	-	−1.4	[−1.56;−1.25]	<0.0001
**HbA_{1c}, mmol/mol**	**227**	**218**	**-**	**-**	**-**
Observed mean at baseline	-	-	68.8	-	-
Estimated mean at week 30	-	-	54.4	-	-
Change from baseline to week 30, %-point	-	-	−15.3	[−17.04;−13.65]	<0.0001
**On-treatment sensitivity analysis**					
**HbA_{1c}, %**	**227**	**210**	**-**	**-**	**-**
Observed mean at baseline	-	-	8.4	-	-
Estimated mean at week 30	-	-	7.1	-	-
Change from baseline to week 30, %-point	-	-	−1.4	[−1.59;−1.27]	<0.0001
**HbA_{1c}, mmol/mol**	**227**	**210**	**-**	**-**	**-**
Observed mean at baseline	-	-	68.7	-	-
Estimated mean at week 30	-	-	54.0	-	-
Change from baseline to week 30, %-point	-	-	−15.6	[−17.34;−13.87]	<0.0001

Response and change in response from baseline analysed using baseline, T2D duration, age, BMI, time, time-squared, pre-initiation use of GLP-1RA, pre-initiation use of DPP-4i, pre-initiation use of insulin, number of OADs used pre-initiation (0–1/2+) and sex as covariates with random intercept and time (slope) for each patient. In-study period represents the time period during which patients are considered to be in the study, regardless of semaglutide treatment status. On-treatment period is the time period in which patients are considered treated with semaglutide. P-value is reported for no average change in response from baseline to week 30.

BMI, body mass index; CI, confidence interval; DPP-4i, dipeptidyl peptidase 4 inhibitor; EOS, end of study; FAS, Full Analysis Set; GLP-1RA, glucagon-like peptide-1 receptor agonist; N, total number of patients in FAS; n, total number of patients included in analyses; OAD, oral antihyperglycaemic drug; T2D, type 2 diabetes.

Supplementary Table S3: ***Post hoc*** **sensitivity analysis of Hba_{1c} and body weight (change from baseline to EOS) in patients that had an EOS visit within the original visit window (weeks 28–38) (EAS)**

	N	n	Estimate	95% CI	P-value
**HbA_{1c}, %**	**189**	**181**	**-**	**-**	**-**
Observed mean at baseline	-	-	8.4	-	-
Estimated mean at EOS	-	-	7.1	-	-
Change from baseline to EOS	-	-	−1.3	[−1.50;−1.16]	<0.0001
**HbA_{1c}, mmol/mol**	**189**	**181**	**-**	**-**	**-**
Observed mean at baseline	-	-	68.2	-	-
Estimated mean at EOS	-	-	53.7	-	-
Change from baseline to EOS	-	-	−14.6	[−16.4; −12.71]	<0.0001
**Body weight, kg**	**189**	**187**	**-**	**-**	**-**
Observed mean at baseline	-	-	99.0	-	-
Estimated mean at EOS	-	-	93.3	-	-
Change from baseline to EOS	-	-	−5.6	[−6.34;−4.93]	<0.0001
Change from baseline to EOS, %-point	-	-	−5.7	[−6.39;−4.97]	<0.0001

ANCOVA adjusted model for response at EOS and change in response from baseline to EOS analysed using baseline, T2D duration, age, BMI, pre-initiation use of GLP-1RA, pre-initiation use of DPP-4i, pre-initiation use of insulin, number of OADs used pre-initiation
(0–1/2+) and sex as covariates. P-value is reported for no average change in response from baseline to EOS. Standard deviation reported.
ANCOVA, analysis of covariance; BMI, body mass index; CI, Confidence interval; DPP-4i, dipeptidyl peptidase 4 inhibitor; EAS, Effectiveness Analysis Set; EOS, End of study;
GLP-1RA, glucagon-like peptide-1 receptor agonist; N, total number of patients in EAS;

n, total number of patients included in analyses; OAD, oral antihyperglycaemic drug; T2D, type 2 diabetes.

Supplementary Figure S1: Change from baseline to EOS in (a) HbA_{1c}; (b) body weight; and (c) waist circumference (EAS)

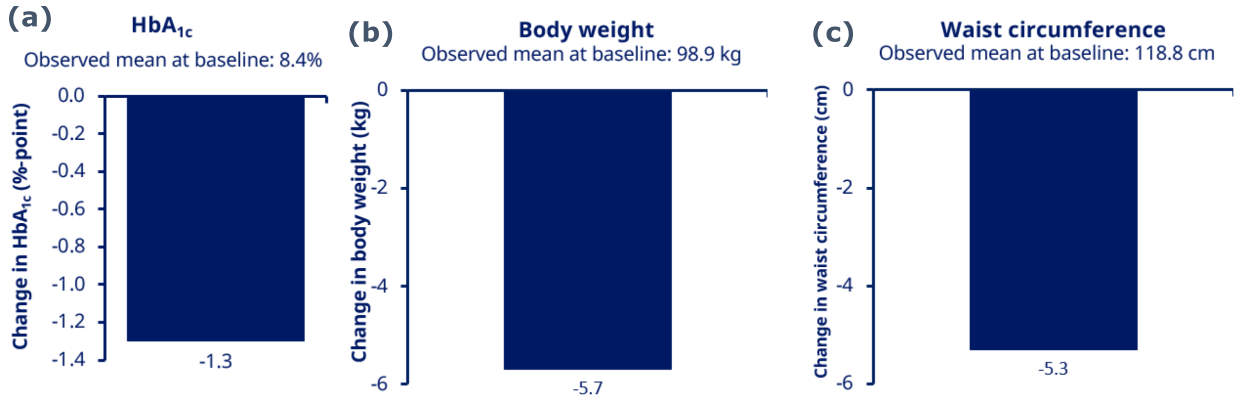

ANCOVA adjusted models for response at EOS and change in response from baseline to EOS is analysed using baseline, T2D duration, age, BMI, pre-initiation use of GLP-1RA, pre-initiation use of DPP-4i, pre-initiation use of insulin, number of OADs used pre-initiation (0–1/2+) and sex as covariates. Crude models, with baseline body weight/waist circumference/HbA_{1c} as the only covariates, produced similar results.

ANCOVA, analysis of covariance; BMI, body mass index; CI, confidence interval; DPP-4i, dipeptidyl peptidase 4 inhibitor; EAS, Effectiveness Analysis Set; EOS, end of study; GLP-1RA, glucagon-like peptide-1 receptor agonist; OAD, oral antihyperglycaemic drug; T2D, type 2 diabetes.

Supplementary Figure S2: Mean HbA_{1c} over time (FAS)

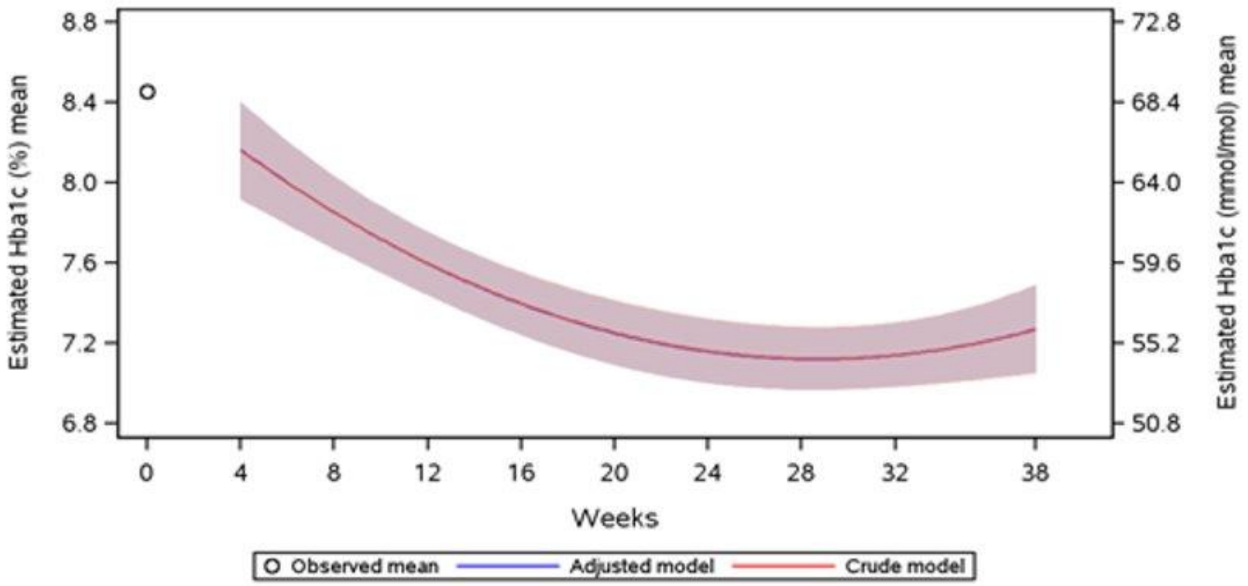

A week 0, observed mean at baseline of patients having at least one post-baseline assessment is plotted. Crude model: HbA_{1c} response is analysed using baseline HbA_{1c}, time and time-squared as covariate with random intercept and time. Adjusted model: HbA_{1c}, T2D duration, age, BMI, time, time-squared, pre-initiation use of GLP-1RA, pre-initiation use of DPP-4i, pre-initiation use of insulin, number of OADs use pre-initiation (0–1/2+) and sex as covariates with random intercept and time. The outlines of the band represent 95% confidence interval.

BMI, body mass index; DPP-4i, dipeptidyl peptidase 4 inhibitor; FAS, Full Analysis Set;
GLP-1RA, glucagon-like peptide-1 receptor agonist; OAD, oral antihyperglycaemic drug; T2D, type 2 diabetes.
